# Indocyanine green fluorescence imaging during laparoscopic rectal cancer surgery could reduce the incidence of anastomotic leakage: a single institutional retrospective cohort study

**DOI:** 10.1186/s12957-022-02856-z

**Published:** 2022-12-13

**Authors:** Akihiro Kondo, Kensuke Kumamoto, Eisuke Asano, Dongping Feng, Hideki Kobara, Keiichi Okano

**Affiliations:** 1 Department of Gastroenterological Surgery, Faculty of Medicine, Kagawa University, 1750-1 Ikenobe, Miki-Cho, Kita-Gun, Kagawa, 761-0793 Japan; 2 Department of Gastroenterology and Neurology, Kagawa University, Miki-Cho, Kagawa, Japan

**Keywords:** Anastomotic leakage, Laparoscopic surgery, Rectal cancer, Indocyanine green, Near-infrared fluorescence imaging

## Abstract

**Background:**

There is insufficient evidence on whether indocyanine green (ICG) fluorescence angiography can reduce the incidence of anastomotic leakage (AL). This retrospective cohort study aimed to evaluate the effect of ICG fluorescence angiography on AL rates in laparoscopic rectal cancer surgery at a single institution.

**Methods:**

Patients who underwent laparoscopic low anterior resection or intersphincteric resection with ICG fluorescence angiography (ICG group; *n* = 73) and patients who underwent a similar surgical procedure for rectal cancer without ICG fluorescence (non-ICG group; *n* = 114) were enrolled consecutively in this study. ICG fluorescence angiography was performed prior to transection of the proximal colon, and anastomosis was performed with sufficient perfusion using ICG fluorescence imaging. AL incidence was compared between both groups, and the risk factors for AL were analyzed.

**Results:**

AL occurred in 3 (4.1%) and 14 (12.3%) patients in the ICG and non-ICG groups, respectively. In the ICG group, the median perfusion time from ICG injection was 34 s, and 5 patients (6.8%) required revision of the proximal transection line. None of the patients requiring revision of the proximal transection line developed AL. In univariate analysis, longer operating time (odds ratio: 2.758; 95% confidence interval: 1.023–7.624) and no implementation of ICG fluorescence angiography (odds ratio: 3.266; 95% confidence interval: 1.038–11.793) were significant factors associated with AL incidence, although the creation of a diverting stoma or insertion of a transanal tube was insignificant.

**Conclusion:**

ICG fluorescence angiography was associated with a significant reduction in AL during laparoscopic rectal cancer surgery. Changes in the surgical plan due to ICG fluorescence visibility may help improve the short-term outcomes of patients with rectal cancer.

## Background

Anastomotic leakage (AL) is a critical complication of colorectal surgery. Previous studies have reported that AL in colorectal surgery occurs in 2–28% of patients [1–7]. AL can worsen short-term and long-term outcomes, such as local recurrence rates [8]. The risk factors of AL have been reported to be male sex, preoperative chemoradiotherapy, and lower anastomosis [9, 10]. In contrast, anastomotic tension, incomplete anastomosis, and vascular perfusion in anastomosis are considered surgery-related factors affecting AL incidence [11–13].

Regarding vascular perfusion in colorectal anastomosis, near-infrared fluorescence imaging using indocyanine green (ICG) has recently been applied to assess intestinal blood flow [14]. The results of the PILLAR II trial showed that the rate of AL was 1.4% in left-sided colorectal resections. Several studies have shown that ICG fluorescence angiography could achieve a low AL prevalence of 2.8 − 4.7% in rectal surgery [15–18]. To our knowledge, only two multicenter randomized controlled trials (RCTs) have investigated the effectiveness of ICG fluorescence angiography on anastomotic leakage after rectal surgery [19, 20]. However, neither trial showed the effectiveness of ICG fluorescence angiography compared with the control group. Therefore, there is insufficient evidence on whether ICG fluorescence can reduce AL incidence.

This retrospective exploratory cohort study aimed to evaluate the effect of ICG fluorescence angiography on AL rates in laparoscopic rectal cancer surgery at a single institution.

## Methods

### Study design

This retrospective exploratory cohort study was conducted at Kagawa University Hospital in Kagawa, Japan. The study was approved by the Institutional Review Board (IRB) of Kagawa University, Kagawa, Japan (approval number: 2021–032). The study and the manuscript adhered to the STROBE guidelines for observational studies. The requirement for informed consent was waived with permission from the IRB due to the retrospective nature of the study.

### Patients

This study included 252 patients who underwent elective laparoscopic surgery for primary rectal adenocarcinoma between January 2014 and December 2021 at our department. Of these patients, we excluded those who underwent abdominoperineal resection or the Hartmann procedure (*n* = 33), or multivisceral organ resection due to tumor invasion (*n* = 25), or who had a history of rectal cancer surgery (*n* = 7). Consequently, 187 patients who underwent laparoscopic anterior resection or intersphincteric resection (ISR) for primary rectal cancer were included in the analysis. Of the 187 patients, 73 who underwent elective laparoscopic anterior resection or ISR with lymphadenectomy for rectal cancer using ICG fluorescence angiography between May 2019 and December 2021 were included. A total of 114 patients who underwent elective laparoscopic anterior resection or ISR with lymphadenectomy for rectal cancer without ICG fluorescence angiography between January 2014 and April 2019 were also included in the control group. During the total study period, surgical procedures and postoperative care were identical before and after ICG use.

### Surgical procedure

The laparoscopic surgical technique was standardized at our institution, and all procedures were performed by experienced colorectal surgeons. After pneumoperitoneum and placement of the five ports, medial to lateral dissection and lymphadenectomy were performed. After dissection of the mesorectum, the rectum was transacted using a linear stapler. Anastomosis for anterior resection was performed using a double-stapling technique with a circular stapler. When performing ISR, hand-sewn anastomosis was performed.

### ICG fluorescence angiography

**Fig. 1:**
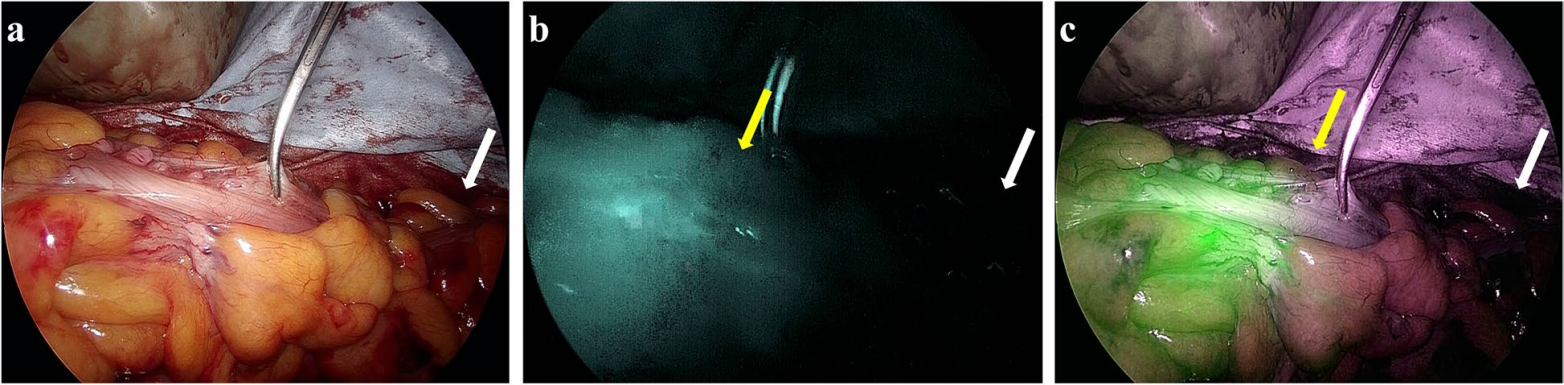
ICG fluorescence imaging. A patient who had the revision of proximal transection line by the findings of ICG fluorescence imaging. **a** Prior to transection of the proximal colon, ICG was injected intravenously, and perfusion assessment was performed. The white arrow indicates the level of planned transection. **b** and **c** No blood perfusion was observed at the level of planned transection (white arrow). The transection line of the proximal colon was changed to the level of sufficient vascular perfusion (yellow arrow)

After rectal transection, the specimen was extracted through the umbilical port, which was extended to approximately 3–5 cm. The concentration of the ICG solution used was 2.5 mg/mL. A bolus of ICG (10.0 mg), followed by a bolus of saline (20 mL), was injected intravenously after dissection of the mesocolon and immediately before transection of the proximal colon. The visualization of ICG fluorescence at the level of the planned transection line, where surgeons choose under macroscopic inspection, was assessed in a completely dark operating room using an Olympus Medical Imaging Video System and Laparoscope (Olympus, Leiderdorp, the Netherlands). Bowel perfusion was judged to be good when ICG fluorescence was identified in the proximal colon serosal face of the planned transection line within 60 s. If the surgeon judged that bowel perfusion was poor via ICG fluorescence imaging, the resection line of the proximal colon was changed to the proximal colon where ICG fluorescence was clearly visible (Fig. 1).

### Data collection

Data pertaining to the following variables were collected and analyzed: patient characteristics, comorbidities, any preoperative therapy (chemoradiotherapy or chemotherapy), surgical procedures, operative time, blood loss, changes in the transection line from the initial part, and postoperative complications, including AL and mortality. Tumor staging was performed using the Union for International Cancer Control tumor-node-metastasis classification system.

### Statistical analyses

Because of the exploratory nature of this study, the sample size was not calculated. Among patients who underwent laparoscopic anterior resection or ISR (*n* = 187), variables were compared between the ICG (*n* = 73) and non-ICG (*n* = 114) groups. Quantitative data were reported as medians (interquartile ranges). Since the data could not assume a normal distribution, the Kruskal–Wallis test was used to compare continuous variables. The chi-square and Fisher’s exact tests were used to compare categorical variables and proportions. Univariate analysis of all patients (*n* = 187) was performed to assess the factors associated with AL. All statistical analyses were performed using JMP statistical software (version 15.1; SAS Institute Inc., Cary, NC, USA). *p*-values < 0.05 were considered statistically significant.

## Results

### Patient characteristics

The patient and tumor characteristics are presented in Table 1. Age, sex, body mass index, and the presence of preoperative comorbidities were not significantly different between both groups. Neoadjuvant treatment (chemoradiotherapy or chemotherapy) was administered more frequently in the ICG group (*p* = 0.01) than in the non-ICG group. Tumor location and preoperative staging were not significantly different between both groups.

**Table 1:** Patient and tumor characteristics

	ICG group (*n* = 73)	Non-ICG group (*n* = 114)	*p*-value
Sex, *n* (%)			0.524
Male	46 (63%)	77 (67.5%)	
Female	27 (37%)	37 (32.5%)	
Age, years^a^	67 (57, 72)	68 (59, 73)	0.517
BMI^a^	22.7 (20.7, 25.1)	22.2 (19.9, 24.8)	0.371
Comorbidities, *n* (%)			
Hypertension	22 (30.1%)	36 (31.6%)	0.835
Diabetes	18 (24.6%)	17 (14.9%)	0.096
Cardiac dysfunction	9 (12.3%)	15 (13.2%)	0.868
Pulmonary dysfunction	9 (12.3%)	10 (8.8%)	0.432
Preoperative therapy, *n* (%)			0.010
Chemoradiotherapy	8 (10.9%)	3 (2.6%)	
Chemotherapy	5 (6.9%)	2 (1.8%)	
Tumor size, mm^a^	30 (20, 50)	35 (20, 50)	0.160
Tumor location, *n* (%)			0.478
Upper rectum	35 (47.9%)	47 (41.2%)	
Middle rectum	8 (11%)	19 (16.7%)	
Low rectum	30 (41.1%)	48 (42.1%)	
Tumor staging, *n* (%)			
Stage I	21 (28.7%)	33 (28.9%)	
Stage II	18 (24.6%)	27 (23.7%)	
Stage III	25 (34.2%)	40 (35.1%)	
Stage IV	9 (12.5%)	14 (12.3%)	

*ICG* indocyanine green, *BMI* body mass index

^a^Values are shown as median (interquartile range)

### Surgical outcomes and ICG fluorescent imaging

Surgical outcomes are presented in Table 2. There were no significant differences in the proportion of operative procedures (high anterior resection, low anterior resection, or ISR) and lateral pelvic lymph node dissection between the two groups. Operating time was significantly shorter in the ICG group (median, 280 min) than in the non-ICG group (median, 333 min) (*p* = 0.008). Although the proportion of diverting stoma creation was not significantly different between both groups, the transanal tube was inserted more often in the ICG group (61%) than in the non-ICG group (27%), which was significantly different (*p* < 0.001). In the ICG group, 5 of 73 patients (6.8%) had the transection line changed to a more proximal colon because blood perfusion at the initially planned bowel was judged to be poor based on ICG fluorescence (Table 2, Fig. 1). One patient required additional bowel resection of 14 cm and underwent splenic flexural mobilization for safe anastomosis.

**Table 2:** Surgical outcomes and ICG fluorescence imaging

	ICG group (*n* = 73)	Non-ICG group (*n* = 114)	*p*-value
Surgical procedure, *n* (%)			0.316
HAR	18 (24.7%)	33 (28.9%)	
LAR	45 (61.6%)	73 (64%)	
ISR	10 (13.7%)	8 (7.1%)	
Lateral lymph node dissection, *n* (%)	8 (10.9%)	18 (15.8%)	0.351
Operative time, min^a^	280 (212, 375)	333 (268, 441)	0.008
Blood loss, mL^a^	11 (0, 81)	24 (0, 114)	0.638
Diverting stoma, n (%)	31 (42.4%)	40 (35.1%)	0.310
Transanal tube, n (%)	45 (61.6%)	31 (27.3%)	< 0.001
The perfusion time after ICG injection, second^b^	34 (11, 82)	−	−
Revision of proximal transection line, *n* (%)	5 (6.8%)	−	−
Distance from the initially planned transection line, cm^b^	8 (3, 14)	−	−

*HAR* high anterior resection, *LAR* low anterior resection, *ISR* intersphincteric resection, *ICG* indocyanine green

^a^Values are shown as median (interquartile range)

^b^Values are shown as median (range)

### Postoperative outcomes

Details of the postoperative complications are presented in Table 3. Seventeen patients were diagnosed with AL. The AL rate was significantly lower in the ICG group (4.1%) than in the non-ICG group (12.3%) (*p* = 0.046). In the ICG group, AL did not occur in cases with a changing transection line due to the ICG fluorescence visibility. The rates of other complications, such as surgical site infection, ileus, urinary dysfunction, and anastomotic bleeding, were not significantly different between both groups. The length of postoperative hospital stay was significantly shorter in the ICG group (median, 11 days) than in the non-ICG group (median, 12 days) (*p* = 0.015). Characteristics of patients diagnosed with AL are presented in Table 4. The AL rates were higher in male patients than in female patients. Operating times were longer in patients diagnosed with AL than in the entire cohort. More than half of patients required reoperation with diverting stoma in both groups. The duration from initial surgery to AL occurrence was shorter in non-ICG group (median, 3 days) than in ICG group (median, 6 days).

**Table 3:** Postoperative outcomes

	ICG group (*n* = 73)	Non-ICG group (*n* = 114)	*p*-value
Anastomotic leakage, *n* (%)	3 (4.1%)	14 (12.3%)	0.046
Surgical site infection, *n* (%)	2 (2.7%)	4 (3.5%)	0.771
Ileus, *n* (%)	4 (5.5%)	13 (11.4%)	0.124
Urinary dysfunction, *n* (%)	8 (10.9%)	13 (11.4%)	0.124
Anastomotic bleeding, *n* (%)	4 (5.5%)	3 (2.6%)	0.273
Reoperation within postoperative 30 days, *n* (%)	3 (4.1%)	9 (7.9%)	0.302
Mortality within postoperative 30 days, *n* (%)	0	0	
Length of postoperative hospital stay, days^a^	11 (10, 14)	12 (9, 18)	0.015

*ICG* indocyanine green

^a^Values are shown as median (interquartile range)

**Table 4:** Characteristics of patients with anastomotic leakage

	ICG group (*n* = 3)	Non-ICG group (*n* = 14)
Sex, *n* (%)		
Male	3 (100%)	11 (78.5%)
Female	0	3 (21.5%)
Age, years^a^	65 (49, 74)	65 (35, 76)
Low rectal cancer	1 (33.3%)	10 (71.4)
Operating time, min^a^	367 (225, 399)	497 (269, 676)
Clavien-Dindo classification ≥ grade 3b	2 (66.6%)	8 (57.1%)
Days from initial surgery to AL occurrence^a^	6 (5, 7)	3 (1, 7)

*ICG* indocyanine green, *AL* anastomotic leakage

^a^Values are shown as median (range)

### Univariate analysis

The results of univariate analysis of the variables associated with the AL incidence are presented in Table 5. The proportion of AL in male patients was higher than that in female patients (11.4% vs. 4.7%), and the proportion of AL in patients with low rectal cancer was higher than that in patients with upper or middle rectal cancer (14.1% vs. 5.5%); however, these differences were insignificant. There were no significant differences in the rates of AL between patients with and without transanal tube insertion (9.2% vs. 9%) and diverting stoma (10.3% vs. 7%). The non-implementation of ICG fluorescence angiography (odds ratio: 3.266; 95% confidence interval: 1.038–11.793) and a longer operating time (odds ratio: 2.758; 95% confidence interval: 1.023–7.624) were significantly associated with a higher AL incidence.

**Table 5:** Univariate analysis of variables associated with the incidence of anastomotic leakage

		AL ( +)	*p*-value	*OR (95% CIs)*
Sex	Male	14/123 (11.4%)	0.083	2.611 (0.821–9.447)
	Female	3/64 (4.7%)		
Age, years	≥ 70	4/70 (5.7%)	0.210	0.484 (0.151–1.550)
	< 70	13/117 (11.1%)		
BMI	> 25	2/27 (7.4%)	0.394	0.506 (0.106–2.419)
	< 25	12/88 (13.6%)		
Tumor location	Low	11/78 (14.1%)	0.051	2.818 (0.994–7.983)
	Upper, middle	6/109 (5.5%)		
Tumor staging	Stages I and II	11/99 (11.1%)	0.312	1.708 (0.604–4.829)
	Stages III and IV	6/88 (6.8%)		
Neoadjuvant therapy	+	2/18 (11.1%)	0.754	1.283 (0.268–6.123)
	−	15/169 (8.9%)		
Surgical procedure	LAR, ISR	14/136 (10.3%)	0.330	1.836 (0.504–6.675)
	HAR	3/51 (5.9%)		
Operating time (min)	≥ 360	10/68 (14.7%)	0.048	2.758 (1.023–7.624)
	< 360	7/119 (5.9%)		
Blood loss (mL)	≥ 100	6/52 (11.5%)	0.472	1.470 (− 0.514–4.204)
	< 100	11/135 (8.2%)		
Transanal tube	−	10/111 (9%)	0.962	0.975 (0.354–2.688)
	+	7/76 (9.2%)		
Diverting stoma	−	12/116 (10.3%)	0.448	1.523 (0.513–4.520)
	+	5/71 (7%)		
ICG fluorescence imaging	−	14/114 (12.3%)	0.046	3.266 (1.038–11.793)
	+	3/73 (4.1%)		

*BMI* body mass index, *ICG* indocyanine green, *HAR* high anterior resection, *LAR* low anterior resection, *ISR* intersphincteric resection, *AL* anastomotic leakage, *OR* odds ratio, *CI* confidence interval

## Discussion

This retrospective cohort study revealed that ICG fluorescence angiography effectively prevents AL after laparoscopic rectal cancer surgery. Furthermore, our results suggested that ICG fluorescence imaging can reduce AL occurrence by utilizing the results of blood perfusion using ICG fluorescence.

In this study, intestinal perfusion was assessed using ICG fluorescence angiography. Traditionally, the presence of blood flow is evaluated using several clinical signs, such as palpable pulsation, peristaltic movement, or active bleeding from the marginal artery [21]. However, these assessment methods were dependent on the surgeons. Karliczek et al. reported that the clinical judgment of surgeons appeared to have low sensitivity and specificity in predicting anastomotic leakage in colorectal anastomoses [22]. Recently, several techniques, such as oxygen spectrometry, laser speckle imaging, thermography, and handheld vital microscopy, have been developed to evaluate intestinal perfusion [23–26]. However, these techniques are not yet widely used due to their high cost and technical complexity. ICG fluorescence angiography was first reported to be useful in colorectal surgery by Kudszus et al. [27]. Trastulli et al. reported that using ICG fluorescence angiography led to a significant reduction in AL in colorectal surgery in a meta-analysis of 25 studies [28]. Moreover, in a meta-analysis of 13 studies involving patients with rectal cancer, Li et al. reported that the use of ICG has a favorable effect on the reduction of the rate of AL [29]. Considering the low cost of ICG dye in Japan (US $6), ICG fluorescence angiography is the most convenient and cost-effective method to evaluate intestinal perfusion.

Regarding AL proportions, we observed a significant reduction in the proportion of AL in the ICG group (4.1%) than in the non-ICG group (12.3%). Previous studies showed that the rates of AL when using ICG fluorescence were 0–9% [15–18, 21, 30–32], similar to our result. Although several studies, including meta-analyses or propensity-score-matched studies, showed the efficacy of this technique in reducing AL incidence [15–18, 28, 29, 31], no RCTs demonstrated this finding [19, 20]. It has been suggested that RCTs might have some flaws, such as sample size and endpoint selection [33]. However, several RCTs are currently ongoing to prove the clinical benefit of routine use of ICG fluorescence [34, 35], such as Essential Trial, whose results remain unpublished. In this study, while operating time was significantly associated with AL incidence after laparoscopic rectal cancer surgery, male sex and tumor location tended to be associated with AL. These findings are consistent with those of previous reports [10, 36, 37]. Furthermore, the differences in operating surgeons and study periods, rather than those in patient characteristics or operative difficulty, might explain our finding regarding the operating time being significantly associated with AL incidence. The retrospective nature of the study should also be considered while interpreting this finding. Nonetheless, our findings suggest that ICG fluorescence has a potential benefit in terms of reducing the risk of AL in rectal cancer surgery.

In this study, the surgical plan for transection of the proximal colon was changed in 6.8% (5/73) of the patients in the ICG group. Although AL did not occur in these patients, if AL had occurred in all of them, the proportion of AL would have increased to 11%, similar to the proportion of AL in the non-ICG group (12.3%). Previous studies have shown that revisions of the proximal transection line were observed in 3.1 − 20.9% [15–19, 30–32, 38]. Of the patients with revision of the proximal transection line, AL occurred in 0–16.7%, as reported in previous studies [15–18, 31, 32, 38]. Although poor intestinal perfusion is not the only cause of AL, anastomosis with sufficient blood perfusion can contribute to reducing AL. Furthermore, considering the characteristics of patients diagnosed with AL in this study, the duration from initial surgery to AL occurrence was shorter in the non-ICG group (median, 3 days) than in the ICG group (median, 6 days), which might be a novel insight into the effect of the use of ICG fluorescence. However, as this was retrospective exploratory study, we were unable to conclusively prove this, and a prospective study is warranted. Our findings suggest that ICG fluorescence angiography to evaluate intestinal perfusion is useful for identifying areas with poor vascular perfusion, which may result in early onset of AL, and changes in the surgical plan due to ICG fluorescence visibility could contribute to a safe anastomosis.

This study has several limitations. First, blood perfusion in the distal rectum was not investigated, which might have influenced AL incidence. Second, a selection bias might have occurred because this was a retrospective cohort study that was not randomized or controlled. Third, the ICG dose used in this study was 10 mg. Although no standard dosage of ICG for evaluating intestinal perfusion has yet been established, the visibility of ICG fluorescence might be different due to the height or body weight of patients. Therefore, the results of this study do not provide definitive evidence for the effectiveness of ICG fluorescence imaging in reducing AL. Fourth, the study periods differed between the groups, and because of the relatively long-term study period, the operating surgeons also differed between the groups. This might have influenced the short-term outcomes. Further multi-institutional, randomized, controlled studies, including ongoing studies, should be planned to verify the definitive benefit of ICG fluorescence in reducing the risk of AL incidence in rectal cancer surgery. However, despite these limitations, we believe that our results are still valuable and applicable because consecutive patients who underwent laparoscopic rectal cancer surgery with anastomosis were assessed, and a standard surgical procedure was performed by a single colorectal team at a single institution.

## Conclusions

In conclusion, ICG fluorescence angiography was associated with a significant reduction in AL during laparoscopic rectal cancer surgery. Additionally, changes in the surgical plan due to the visibility of ICG fluorescence may help improve the short-term outcomes of patients with rectal cancer. Although this was a retrospective study, it provides valuable insights into the efficacy of ICG fluorescence in patients who have undergone rectal cancer surgery by comparing the rates of AL between surgeries performed with and without ICG fluorescence. Further prospective large clinical trials are warranted to validate the definitive efficacy of ICG fluorescence imaging in reducing AL after laparoscopic rectal cancer surgery.

## Data Availability

The datasets used and/or analyzed during the current study are available from the corresponding author on reasonable request.

## Abbreviations

AL: Anastomotic Leakage
ICG: Indocyanine Green
IRB: Institutional Review Board
ISR: Intersphincteric Resection
RCT: Randomized Controlled Trial
